# Widowhood Impairs Emotional Cognition Among Elderly

**DOI:** 10.3389/fnagi.2021.808885

**Published:** 2022-01-31

**Authors:** Taiyong Bi, Hui Kou, Yanshu Kong, Boyao Shao

**Affiliations:** Center for Mental Health Research in School of Management, Zunyi Medical University, Zunyi, China

**Keywords:** widowed elderly, cognitive deficit, emotional cognition, attention, visual working memory

## Abstract

**Background:**

The negative impacts of spousal bereavement on the emotional health of the elderly (e.g., depression and anxiety) have been revealed. However, whether widowhood impairs emotional cognition among the elderly is less known. The purpose of this study is to reveal the emotional cognitive deficits among the widowed elderly.

**Methods:**

In this study, we recruited 44 widowed elderly (WE) and 44 elder couples (non-widowed elderly, NWE) and examined their emotional cognition including attention and visual working memory, which were measured by the visual search task and delayed-match-to-sample task, respectively. Three kinds of emotional faces (i.e., sad, angry, and happy) were adopted as the attentional or mnemonic targets.

**Results:**

It revealed that WE had a general deficit in search efficiency across emotional types, while they showed mnemonic deficits in negative faces but not positive faces. Furthermore, the modeling analysis revealed that the level of depression or state anxiety of the elderly moderated the effects of widowhood on the deficits of mnemonic processing, i.e., the deficits were only evident among WE with the high level of depression or state anxiety.

**Conclusion:**

These findings reveal the attentional deficits in sad, angry, and happy faces and the mnemonic deficits in sad and angry faces among elderly who suffer from widowhood and point out the important role of emotional problems such as depression and state anxiety in modulating these emotional cognitive deficits.

## Introduction

Spousal bereavement is a common negative event in later life that negatively influences the mental and physical health of the elderly. The widowed elderly reported increased anxiety, grief, depression, emotional loneliness, and social loneliness (Bergman et al., 2010; Kim and Lyu, 2018; Förster et al., 2019; Szabó et al., 2020). These negative impacts of widowhood were found to last a long period and thus were serious threats to wellbeing and quality of life in the elderly (van Boekel et al., 2019). Although the negative impacts of spousal bereavement on the emotional health of the elderly have been revealed, whether widowhood impairs emotional cognition among the elderly is less known. The purpose of this study is to reveal the emotional cognitive deficits among the widowed elderly.

First of all, emotional cognitions could change with aging. From the perspective of socioemotional selectivity theory, aged ones are motivated to derive emotional meaning from life and adopt an adaptive emotion regulation strategy to maximize the positive affect and minimize the negative affect (Carstensen et al., 1999). Evidence showed cognitive biases to positive emotion among the elderly, e.g., perceiving ambiguous expressions more positive (Kellough and Knight, 2012) and reliable attention to positive stimuli (e.g., happy faces) (Carstensen and Mikels, 2005; Reed et al., 2014; Gronchi et al., 2018). Correspondingly, electrophysiological activities were found higher for pleasant pictures than unpleasant ones among elderly (Pehlivanoglu and Verhaeghen, 2019). In addition to the cognitive bias to positive emotion, the elderly also allocated fewer resources to negative emotions. For example, the elderly showed attentional inhibition and attentional avoidance to negative emotions (e.g., sad and angry faces) (Mather and Carstensen, 2003; Bannerman et al., 2011). Electrophysiological evidence also revealed that angry faces evoked a smaller P1 amplitude in the elderly compared with that in young adults (Mienaltowski et al., 2011). However, it should also be noted that some studies suggested cognitive biases to both positive and negative emotions among the elderly (Smith et al., 2005; Murphy and Isaacowitz, 2008). Furthermore, it was indicated that the attentional bias toward happy faces is an automatic process, and the attentional avoidance of fearful faces is a controlled mechanism (Gronchi et al., 2018). Taken together, aging increases cognitive biases to positive emotions (Carstensen and Mikels, 2005) and possibly decreases the allocation of cognitive resource to negative emotions.

Another factor that may affect emotional cognition could be the emotional state. According to the cognitive-behavioral model, individuals with high anxiety/depression have distorted or dysfunctional cognitive structure-labeled schemas that lead to distorted or biased information processing, which plays a central role in the development and maintenance of depression/anxiety (Beck, 1964, 1967; Rapee and Heimberg, 1997; Turk et al., 2001; Heimberg et al., 2010; Beck and Haigh, 2014). It has been shown that highly anxious individuals showed attentional bias toward threatening faces (e.g., angry faces) (Bradley et al., 1999; Gamble and Rapee, 2010; Liang et al., 2017; Abend et al., 2018), while, individuals with depression showed selective attention to sad faces (Joormann and Gotlib, 2007; Kujawa et al., 2011) and dwell longer on sad faces (Lazarov et al., 2018). Among the aged ones, it also revealed that depressed elderly recognized sad and angry faces better than happy faces (Shiroma et al., 2016; Ferreira et al., 2021), and anxious elderly showed attentional vigilance to sad faces (Lee and Knight, 2009). Therefore, emotional problems such as depression and anxiety may bias the cognition to negative stimuli.

Although the effects of aging and emotional state on emotional cognition have been well studied, the effect of widowhood on emotional cognition among the elderly was rarely examined. On one hand, according to the socioemotional selectivity theory, the elderly might prefer positive emotion. It thus can be hypothesized that the widowed elderly might have cognitive biases toward happy faces. In contrast, according to the cognitive-behavioral model, it can be hypothesized that the widowed elderly might have biased cognition to negative emotions, as the level of anxiety and depression may be higher among them. To reconcile this discrepancy, we conducted behavioral measurements of emotional cognitions between widowed elderly and elder couples. Both attentional processing and working memory processing were examined. Moreover, we explored the moderating roles of depression and anxiety in these cognitive deficits.

Regarding the attentional processing, we adopted the visual search paradigm in which participants were asked to detect, locate, or identify a target among distractors as quickly as possible. The results reveal how attention suppresses irrelevant distractors as well as shifts/orients to the target. The visual search paradigm is a well-established approach in measuring visual attention, which has been pervasively adopted to test emotional attention across adults of different ages (Hahn et al., 2006; Mather and Knight, 2006; Lundqvist et al., 2013; Dodd et al., 2017; Wieser et al., 2018; Suslow et al., 2021). Besides the accuracy and reaction time (RT), the search slope is an important indicator of search efficiency and should also be examined. A search slope is the slope of the fitting line of RT against the size of the search array. A steeper search slope denotes a large increase of the RT with the increase of distractors and inefficiency in searching the target.

Regarding the working memory processing, we adopted the delayed-match-to-sample (DMTS) paradigm, which is one of the most common tasks used to study visual working memory. The DMTS task consists of three phases, namely, a sample (encoding) phase, a delay (maintenance) phase, and a test (retrieval) phase. It is mainly used to examine the accuracy and capacity in encoding and maintaining visual stimuli. Previous studies showed stable test-retest reliability in the DMTS task (Borghans and Princen, 2012) and stable brain structures associated with the task (i.e., dorsolateral prefrontal cortex, fusiform gyrus, and posterior parietal cortex) (Daniel et al., 2016). Most of the studies concerning emotional cognition among the elderly focused on perceptual and attentional processing, while only a few examined mnemonic processing. Nevertheless, working memory processing is also an important aspect of emotional cognition, which is suggested as a core cognitive ability (Jackson et al., 2008, 2009; Jackson et al., 2014; Thomas et al., 2014; Stiernströmer et al., 2016). The capacity of the central executive system in working memory declined with age (Baddeley, 1986), resulting in a lower information-processing capacity among the elderly (Salthouse, 2016). Some studies indicated that emotion enhanced the working memory among both younger and elder adults, regardless of the valence of emotion (Mammarella et al., 2013; Truong and Yang, 2014). However, other studies suggested a positivity superiority in the processing of working memory among the elderly (Mikels et al., 2005; Bermudez and Souza, 2017). Therefore, the effect of emotion on working memory processing of faces among widowed elderly remains to be examined.

Taken together, this study was designed to reveal the emotional cognitive deficits among widowed elderly, by comparing attentional bias and mnemonic bias to emotional stimuli between widowed and non-widowed elderly. Furthermore, we aimed to examine the role of the negative emotional state induced by widowhood in the cognitive deficits among widowed individuals. The results of this study may help test and develop existing theories such as the socioemotional selectivity theory and the cognitive-behavioral model and help develop interventions on the emotional problems among widowed elderly.

## Materials and Methods

### Participants

Forty-four widowed elderly (i.e., 29 women, WE group) and 44 non-widowed elderly (i.e., 22 couples, NWE group) were recruited from southwestern China. The inclusion criteria for the WE group were: (1) older than 55 years; (2) currently widowed and has no partner; and (3) able to understand the contents of the questionnaires and to complete the behavioral tasks. The inclusion criteria for the NWE group were: (1) older than 55 years; (2) currently in marriage and lives with the partner; and (3) the couple could both understand the contents of the questionnaires and complete the behavioral tasks. All of the participants were right-handed and had normal or corrected-to-normal vision and normal color vision. We performed a power analysis to determine the sample size using the G*Power 3.1.9.7 software (Faul et al., 2009). To find a significantly different effect between groups, we set the level of effect size as *d* = 0.8 with a statistical power of 0.9. The result showed that two samples of 34 subjects, at least, were required. The distribution of sex was not significantly different between groups (χ^2^ = 2.28, *p* = 0.131). However, the age of the WE group (*M* = 70.68, *SD* = 7.35, range: 59–87 years old) was significantly larger than that of the NWE group (*M* = 65.86, *SD* = 5.69, range: 55–77 years old) [*t*(86) = 3.44, *p* = 0.001]. Therefore, age was treated as a covariate in all the analyses. In addition, although the sex distribution was not significantly different between groups, to fully exclude the influence of sex, we also treated sex as a covariate in all the analyses. The study was approved by the ethics committee for human research at Zunyi Medical University, and informed consent was obtained from all participants.

### Measurements

All the participants completed two scales before they participated in behavioral experiments.

*The Geriatric Depression Scale* (GDS; Yesavage et al., 1982). The Geriatric Depression Scale (GDS) is designed to measure depression in the elderly (Yesavage, 1988) and has been widely used among the elderly (Durmaz et al., 2018; Shin et al., 2019; Krishnamoorthy et al., 2020). It consists of 30 items, and participants respond to the items by choosing *yes* or *no*. The total score ranges from 0 to 30 and indicates the severity of depression.

*The State-Trait Anxiety Inventory* (STAI; Spielberger et al., 1983). The State-Trait Anxiety Inventory (STAI) can differentiate between the temporary condition of “state anxiety” and the more general and long-standing quality of “trait anxiety” (Spielberger et al., 1983; Spielberger, 2010). In this study, we focused on the impact of spousal bereavement on the mental health of the elderly. Therefore, state anxiety may be more related to our purpose, and the influence of trait anxiety should be excluded. The scale consists of two subscales, namely, STAI-S and STAI-T. The STAI-S subscale consists of twenty items, which assess the state anxiety. The STAI-T subscale also consists of twenty items, which assess the general anxiety as a long-standing trait. The total scores of the two subscales both range from 20 to 80 and indicate the severity of anxiety.

### Experiment 1: The Visual Search Task

#### Stimuli

Sixteen sad faces, sixteen angry faces, and sixteen happy faces were selected from the Chinese Facial Affective Picture System (CFAPS) as search targets. Fifty-eight neutral faces selected from the CFAPS were treated as distractors. Half of the faces were women, and the other half were men. The hair, ears, and necks of all faces were removed using the Photoshop software. We then laid each picture on a black background and cropped it into a uniform size (130 × 150 pixels). All photos were then grayscaled and matched for brightness and contrast using the MATLAB software. The valence and arousal of each face were evaluated on 9-point scales. Valence was significantly different among three categories of emotional faces [*F*(2, 30) = 351.92, *p* < 0.001, η*_*p*_*^2^ = 0.959]. *Post-hoc* tests with Bonferroni adjustment revealed that the valence of happy faces (*M* = 6.74, *SD* = 0.51) was higher than that of sad faces (*M* = 2.84, *SD* = 0.54) and angry faces (*M* = 2.62, *SD* = 0.40) (all *p* < 0.001), while the valence was not different between sad and angry faces (*p* = 0.760). The difference of arousal among the three categories of emotional faces was non-significant [*F*(2, 30) = 1.59, *p* = 0.224,η*_*p*_*^2^ = 0.096].

#### Procedure

The visual stimuli were presented on a SAMSUNG 19-in LCD screen (spatial resolution: 1,280 × 800; refresh rate: 60 Hz), with a Dell PC (CPU: Intel Core i5-4590; Graphics card: Intel HD Graphics 4600; RAM: 4 GB) (Zhang et al., 2018). The subjects viewed the stimuli from a distance of 60 cm. The presentation of stimuli was controlled using the E-Prime 2.0 software.^1^

In a visual search task, participants are asked to find a target among several distractors. In this study, the target was the emotional face (i.e., with happy, sad, or angry expression), while the distractor was the neutral face. At the beginning of each trial, a white fixation cross was presented at the center of the black screen for a random period of 500–1,500 ms. Then, an array of eight faces or two faces appeared until a response was made. In 75% of the trials, the array contained a search target, while in the rest trials, the target was absent. Participants were asked to press one key (F) if they found the target (an emotional face) among the distractors (neutral faces) and another key (J) if the target was absent as quickly and accurately as possible. The next trial began after a blank screen for 500 ms. Each experimental block consisted of 64 trials. The set size (i.e., number of faces in the face array) and search target were fixed in each block. As there were two kinds of set size (two or eight faces) and three kinds of search targets (i.e., sad, angry, or happy faces), each participant was required to complete six experimental blocks of 384 trials. The location of the target and the identity of faces were randomized trial by trial.

#### Designs and Analysis

This experiment was a 3 (emotional type: sad/angry/happy) × 2 (set size: 2/8) × 2 (group: WE/NWE) mixed design, with the emotional type and set size as within-subject factors and the group as a between-subject factor. As discussed in participants, age and sex were treated as covariates in all the analyses.

First, accuracies were calculated and analyzed with a 3 (emotional type) × 2 (set size) × 2 (group) mixed analysis of covariance (ANCOVA). Second, RTs beyond 3 SD from the mean RT were excluded. RTs were then calculated based on remaining trials and analyzed with a 3 (emotional type) × 2 (set size) × 2 (group) mixed ANCOVA. Third, for the visual search paradigm, the search slope is an indicator of search efficiency. The search slope is defined as the slope of the fitted line of RT against set size. In this study, the search slope = (RT_8_–RT_2_)/(8–2). RT_8_ denotes the RT from trials with eight faces, while RT_2_ denotes the RT from trials with two faces. The larger the slope, the lower the efficiency of searching the targets. Search slopes were analyzed with a 3 (emotional type) × 2 (group) mixed ANCOVA.

### Experiment 2: The Delayed-Match-to-Sample Task

#### Stimuli

Seventy-two emotional faces, namely, 24 sad, 24 angry, and 24 happy faces, were selected from CFAPS, half of which were female faces. Images were processed in the same way as Experiment 1, except that the image size was set to 185 × 200 pixels. Repeated measures ANOVA showed that the valences were different among emotional faces [*F*(2, 46) = 632.01, *p* < 0.001,η*_*p*_*^2^ = 0.965]. *Post-hoc* tests suggested that the valence of happy faces (*M* = 6.54, *SD* = 0.64) was significantly higher than those of angry (*M* = 2.70, *SD* = 0.44) and sad (*M* = 2.85, *SD* = 0.48) faces (*ps* < 0.001), while the difference was not significant between sad and angry faces (*p* = 0.490). In addition, no significant difference in arousal was found [*F*(2, 46) = 2.67, *p* = 0.080, η*_*p*_*^2^ = 0.104].

#### Procedure

In a DMTS task, participants were asked to briefly remember a sample stimulus for a few seconds. Then, a test stimulus was presented, and participants compared it with the sample stimulus. In this study, each trial began with a white cross at the center of the black screen for a random period of 500–1,500 ms. Subsequently, two faces (sample) with the same expression appeared for 1,000 ms at two of the four-quadrant of the visual field. Afterward, a blank screen was presented for 2,000 ms, and participants were asked to maintain the two faces they just saw in their minds. Then, a test face was presented at the center of the screen for 1,000 ms. The test face matched the sample face in half of the trials. Subjects were asked to determine whether the test face matched one of the two sample faces (F for yes, J for no). The next trial began after a response was made. Each block included 48 trials and contained faces with one facial emotion. As there were three kinds of emotions, each participant was required to complete three blocks in the experiment. The sequence of blocks was randomized among participants.

#### Designs and Analysis

This experiment was a 3 (emotional type: sad/angry/happy) × 2 (group: WE/NWE) mixed design, with the emotional type as a within-subject factor and group as a between-subject factor. Age and sex were treated as covariates in all the analyses.

First, accuracies were calculated and analyzed with a 3 (emotional type) × 2 (group) mixed ANCOVA. Second, RTs within the 3 SD from the mean RT were calculated and analyzed with a 3 (emotional type) × 2 (group) mixed ANCOVA. Third, we calculated the discriminability index, *d*′, according to the signal detection theory. *d*′ is defined as the distance between the two distributions of signal and noise. In this study, a signal is a face that matched the sample face, while noise is a face that did not match any sample faces. The hit rate and false alarm rate were calculated first. *d*′ was then calculated as *Z*(Hit)-*Z*(False alarm). *Z* is the *Z*-transformed score of a rate. A larger *d*′ denotes a higher discriminability of the subject in identifying matched or unmatched faces.

### Modeling Analysis

To investigate the moderation effects of depression and anxiety on the relationship between widowhood and cognitive processing, we performed the modeling analysis using the PROCESS V3.4.1 plugin in the SPSS 23.0 software.^2^ Moderation models were constructed whenever the between-group difference of cognitive processing was found. In these models, the group was set as the independent variable (0 = NWE, 1 = WE); behavioral performance was set as the dependent variable (e.g., RT in searching sad face among eight items); the GDS score or STAI-S score was set as the moderator; age and sex were set as covariates.

## Results

### The Level of Depression and Anxiety

The demographics and scale scores are summarized in Table 1. The WE group showed a higher depression level and a higher state anxiety level than the NWE group (both *p* < 0.05), while the trait anxiety was not significantly different between the two groups (*p* = 0.560).

**TABLE 1:** Demographics and scale scores [mean (SD)].

	WE (*N* = 44)	NWE (*N* = 44)	*t*/χ^2^	*p*
Age (years)	70.68 (7.35)	65.86 (5.70)	3.44	0.001
Sex (female/male)	29/15	22/22	2.29	0.131
GDS	10.23 (5.87)	7.91 (4.03)	2.16	0.034
STAI-S	36.82 (8.19)	32.89 (5.86)	2.59	0.011
STAI-T	38.84 (7.34)	37.93 (7.17)	0.58	0.560

*WE, widowed elderly; NWE, non-widowed elderly; GDS, Geriatric Depression Scale; STAI-S, State-Trait Anxiety Inventory–State anxiety subscale; STAI-T, State-Trait Anxiety Inventory–Trait anxiety subscale.*

### Experiment 1: The Visual Search Task

Averaged accuracies, RTs, and slopes are presented in Supplementary Table 1.

First, a 3 (emotional type) × 2 (set size) × 2 (group) mixed ANCOVA was performed on the accuracies. No significant interactions and main effects were found (all *F* < 1.9, *p* > 0.1, η*_*p*_*^2^ < 0.03).

Next, the same 3 × 2 × 2 mixed ANCOVA was performed on the RTs. There was a significant interaction between the set size and group[(*F*(1, 84) = 10.99, *p* = 0.001, η*_*p*_*^2^ = 0.116]. The simple effect analysis showed that WE responded more slowly than NWE when the set size was 8 (*p* = 0.003), while the difference was non-significant when the set size was 2 (*p* = 0.406). Furthermore, the interaction between the emotional type and set size was significant [*F*(2, 168) = 3.27, *p* = 0.041, η*_*p*_*^2^ = 0.037]. The simple effect analysis showed that the effect of the emotional type was marginally significant when the set size was 8 (*p* = 0.092), while it was non-significant when the set size was 2 (*p* = 0.800). Specifically, when the set size was 8, RTs to happy faces were faster than those to sad (*p* < 0.001) and angry (*p* < 0.001) faces. In addition, there were significant main effects of set size [*F*(1, 84) = 10.87, *p* = 0.001, η*_*p*_*^2^ = 0.115] and group [*F*(1, 84) = 5.04, *p* = 0.027, η*_*p*_*^2^ = 0.057], indicating faster responses at set size of 2 and in the NWE group. Other interactions and main effects were non-significant (all *F* < 2.3, *p* > 0.1, η*_*p*_*^2^ < 0.03).

Finally, a 3 (emotional type) × 2 (group) mixed ANCOVA was performed on the search slopes. We found a significant main effect of group [*F*(1, 84) = 10.99, *p* = 0.001, η*_*p*_*^2^ = 0.116], indicating lower search efficiencies among WE. Furthermore, the main effect of the emotional type was significant [*F*(2, 168) = 3.27, *p* = 0.041, η*_*p*_*^2^ = 0.037]. *Post-hoc* tests showed a marginally significant difference between the slopes for happy and sad faces (*p* = 0.093). The interaction between the emotional type and the group was non-significant [*F*(2, 168) = 1.53, *p* = 0.219, η*_*p*_*^2^ = 0.018].

### Experiment 2: The Delayed-Match-to-Sample Task

We first performed a 3 (emotional type) × 2 (group) mixed ANCOVA on the accuracies. Results showed that the interaction effect was significant [*F*(2, 168) = 3.69, *p* = 0.027, η*_*p*_*^2^ = 0.042]. The simple effect analysis revealed that WE had lower accuracies in memorizing sad (*p* = 0.071) and angry (*p* = 0.050) faces than NWE, while the difference was non-significant for happy faces (*p* = 0.559). The main effects of emotional type and the group were non-significant (both *F* < 2.2, *p* > 0.1, η*_*p*_*^2^ < 0.03).

Next, a 3 (emotional type) × 2 (group) mixed ANCOVA was performed on the RTs. No significant interaction and main effects were found (all *F* < 1.6, *p* > 0.2, η*_*p*_*^2^ < 0.02).

Finally, a 3 (emotional type) × 2 (group) mixed ANCOVA on the *d*′. Similar to the accuracy results, we found a significant interaction effect between the emotional type and group [*F*(2, 168) = 4.12, *p* = 0.018, η*_*p*_*^2^ = 0.047]. The simple effect analysis revealed lower discriminability among WE in memorizing sad (*p* = 0.071) and angry (*p* = 0.037) faces than NWE, while the difference was non-significant for happy faces (*p* = 0.544). The main effects of the emotional type and the group were non-significant (both *F* < 1.6, *p* > 0.2,η*_*p*_*^2^ < 0.02).

### Modeling Analysis

**FIGURE 1:**
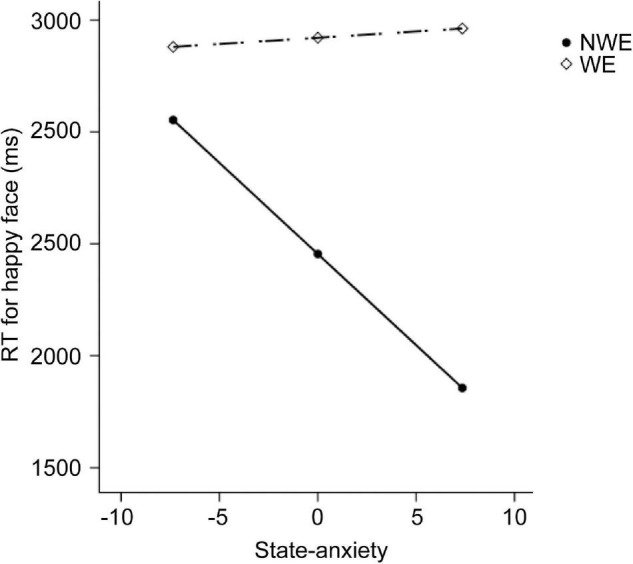
The moderation effect of state anxiety on the relationship between widowhood and the performance in the visual search experiment. The dependent variable here is the reaction time (RT) for happy faces at the set size of 8. NWE, non-widowed elderly; WE, widowed elderly.

First, regarding the visual search task, we found significant between-group differences on the RTs at a set size of 8, as well as the difference on the search slopes. Therefore, six moderation models with depression as moderators and six moderation models with state anxiety as moderators were constructed. As we described in methods, group and behavioral performance were set as the independent variable and dependent variable, respectively. The level of depression or state anxiety was set as the moderator. Age and sex were set as the covariates. The moderation effects of depression and state anxiety are summarized in Tables 2, 3, respectively. The results only showed a significant moderation effect of state anxiety in the relationship between the group and RTs for happy faces (Table 3). Simple slope tests (Figure 1) revealed that NWE showed faster responses to happy faces than WE only among high state anxiety individuals (*b* = 1,605.76, *t* = 3.51, *p* = 0.001, 95%CI [696.30, 2,515.21]), while the difference was non-significant among low state anxiety individuals (*b* = 326.73, *t* = 0.75, *p* = 0.453, 95%CI [–536.63, 1,190.09]).

**TABLE 2:** The interaction effects of group × depression in the visual search task.

Dependent variables	*b*	*SE*	*t*	*p*	95%CI
Search slope (sadness)	–4.94	11.09	–0.45	0.66	[–27.01, 17.13]
Search slope (anger)	–13.54	9.66	–1.40	0.16	[–32.76, 5.67]
Search slope (happiness)	12.67	10.31	1.23	0.22	[–7.85, 33.18]
RT at set size of 8 (sadness)	18.95	76.06	0.25	0.80	[–132.35, 170.25]
RT at set size of 8 (anger)	7.36	71.07	0.10	0.92	[–134.02, 148.75]
RT at set size of 8 (happiness)	114.24	61.02	1.87	0.06	[–7.15, 235.62]

**TABLE 3:** The interaction effects of group × state anxiety in the visual search task.

Dependent variables	*b*	*SE*	*t*	*p*	95%CI
Search slope (sadness)	5.22	7.79	0.67	0.50	[–10.28, 20.71]
Search slope (anger)	–8.28	6.83	–1.21	0.23	[–21.87, 5.32]
Search slope (happiness)	7.58	7.29	1.04	0.30	[–6.91, 22.08]
RT at set size of 8 (sadness)	89.12	52.50	1.70	0.09	[–15.32, 193.56]
RT at set size of 8 (anger)	46.74	49.47	0.94	0.35	[–51.67, 145.15]
RT at set size of 8 (happiness)	87.04	42.97	2.03	**0.04**	[1.56, 172.51]

*Bold values denote significant statistical results with p values < 0.05.*

**FIGURE 2:**
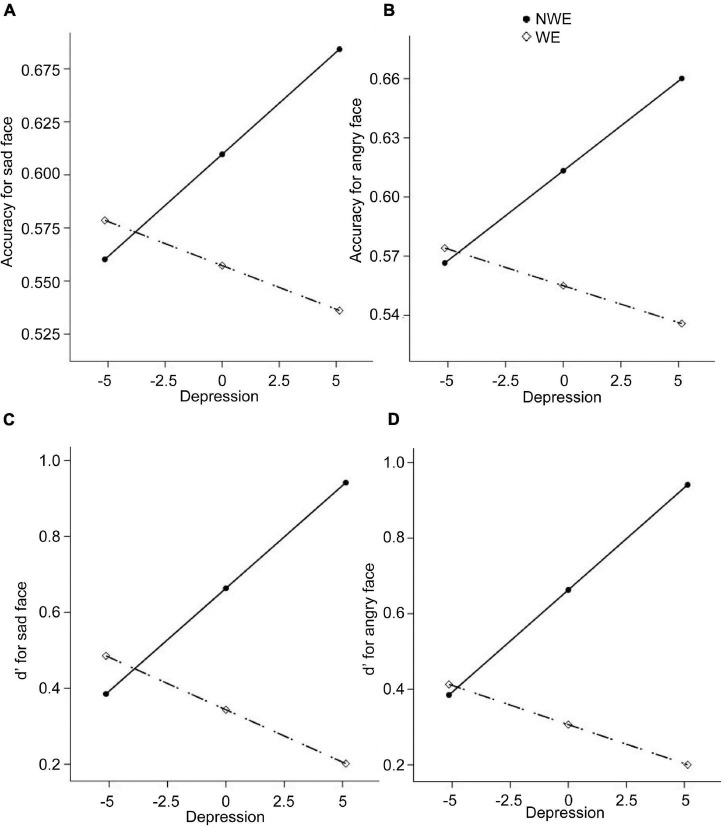
The moderation effect of depression on the relationship between widowhood and the performance in the delayed-match-to-sample (DMTS) experiment. The dependent variables are the mnemonic accuracy for sad faces, the mnemonic accuracy for angry faces, *d*′ for sad faces, and *d*′ for angry faces, respectively, for **(A–D)**. NWE, non-widowed elderly; WE, widowed elderly.

**FIGURE 3:**
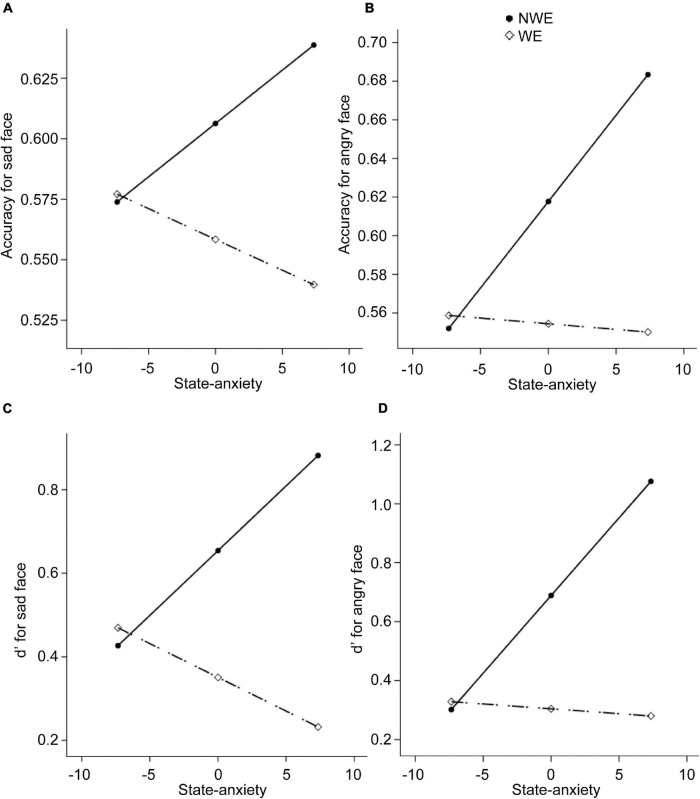
The moderation effect of state anxiety on the relationship between widowhood and the performance in the DMTS experiment. The dependent variables are the mnemonic accuracy for sad faces, the mnemonic accuracy for angry faces, *d*′ for sad faces, and *d*′ for angry faces, respectively, for **(A–D)**. NWE, non-widowed elderly; WE, widowed elderly.

Next, regarding the DMTS task, we found significant between-group differences in the accuracies and *d*′ for sad and angry faces. Therefore, four moderation models with depression as moderators and four moderation models with state anxiety as moderators were constructed. Results are summarized in Tables 4, 5. All the moderation effects were significant. The simple slope analysis (Figure 2) on the effect of depression revealed that NWE showed a higher mnemonic accuracy and discriminability than WE only among high-depressive individuals (accuracies for sad faces: *b* = –0.12, *t* = –3.40, *p* = 0.001, 95%CI [–0.19, –0.05]; accuracies for angry faces: *b* = –0.12, *t* = –3.25, *p* = 0.002, 95%CI [–0.20, –0.05]; *d*′ for sad faces: *b* = –0.74, *t* = –3.25, *p* = 0.002, 95%CI [–1.19, –0.29]; *d*′ for angry faces: *b* = –0.74, *t* = –3.39, *p* = 0.001, 95%CI [–1.18, –0.31]) but not among low-depressive individuals. Similar results were found on the effect of state anxiety (Figure 3). NWE showed a higher mnemonic accuracy and discriminability than WE only among high state anxiety individuals (accuracies for sad faces: *b* = –0.10, *t* = –2.70, *p* = 0.008, 95%CI [–0.17, –0.03]; accuracies for angry faces: *b* = –0.74, *t* = –3.61, *p* = 0.001, 95%CI [–0.21, –0.06]; *d*′ for sad faces: *b* = –0.65, *t* = –2.85, *p* = 0.006, 95%CI [–1.10, –0.20]; *d*′ for angry faces: *b* = –0.80, *t* = –3.78, *p* < 0.001, 95%CI [–1.21, –0.38]) but not among low state anxiety individuals.

**TABLE 4:** The interaction effects of group × depression in the DMTS task.

Dependent variables	*b*	*SE*	*t*	*p*	95%CI
Accuracies (sadness)	–0.01	0.004	–2.88	**0.01**	[–0.02, –0.004]
Accuracies (anger)	–0.01	0.01	–2.54	**0.01**	[–0.02, –0.002]
d′ (sadness)	–0.08	0.03	–2.72	**0.01**	[–0.14, –0.02]
d′ (anger)	–0.07	0.03	–2.60	**0.01**	[–0.13, –0.02]

*Bold values denote significant statistical results with p values < 0.05.*

**TABLE 5:** The interaction effects of group × state anxiety in the delayed-match-to-sample (DMTS) task.

Dependent variables	*b*	*SE*	*t*	*p*	95%CI
Accuracies (sadness)	–0.01	0.003	–2.02	**0.04**	[–0.01, –0.0001]
Accuracies (anger)	–0.01	0.004	–2.74	**0.01**	[–0.02, –0.003]
d′ (sadness)	–0.05	0.02	–2.20	**0.03**	[–0.09, –0.01]
d′ (anger)	–0.06	0.02	–2.83	**0.01**	[–0.10, –0.02]

*Bold values denote significant statistical results with p values < 0.05.*

## Discussion

In this study, we examined the effect of spousal bereavement on the emotional state and emotional cognition among the elderly. First, we found that WE indeed showed a higher level of depression and state anxiety than NWE, consistent with previous studies (Williams, 2005; Ward et al., 2007; Sikorski et al., 2014; Tseng et al., 2017; Verma et al., 2019). Second, accompanied by these emotional problems, emotional cognitions were impaired. Specifically, a general deficit in visual search efficiency was observed among WE. However, the deficits in visual working memory were restricted to negative emotions (i.e., sadness and anger). We further examined the moderating effects of emotional problems on the relationship between spousal bereavement and emotional cognition deficits. The results mainly showed that emotional problems could modulate the effect of spousal bereavement on working memory deficits, i.e., the deficits were only evident among individuals with a high level of emotional problems. These results demonstrated different mechanisms between the deficits in attentional processing and mnemonic processing among WE.

In general, our results revealed cognitive deficits among WE, consistent with previous findings (S.H. Shin et al., 2018). After controlling for the effects of depression, social vulnerability, and stress, widowhood still showed a negative influence on the cognitive health of elderly (Zhao et al., 2021). The core of cognitive abilities is mainly limited by information-processing capacity, which decreases with age (Lavie, 2005; Salthouse, 2016). Previous studies suggested that widowed elderly people showed declined information-processing speed (Ward et al., 2007; Atalay and Staneva, 2020). Our results also suggest that WE had declined information-processing capacity. For example, when the task requirement was relatively low (e.g., only one distractor in the visual search task), WE performed equally well as NWE.

Although the general deficit was found on emotional cognition, the effects of the emotional valance on these deficits may be different between specific tasks. For the attentional processing, the deficits seemed not dependent on the emotional valence. It seemed to support neither the socioemotional selectivity theory nor the cognitive-behavioral model. According to the socioemotional selectivity theory (Carstensen et al., 1999), all elderly should have attentional biases toward happy faces. And according to the cognitive-behavioral model (Beck, 1964, 1967; Rapee and Heimberg, 1997; Turk et al., 2001; Heimberg et al., 2010; Beck and Haigh, 2014), the WE should have attentional biases toward sad faces with their increasing level of anxiety and depression. A potential reason may be that the deficits in attentional processing might stem from inhibitory deficits. According to the inhibitory deficit theory of cognitive aging, the ability to inhibit irrelevant information in selective attention declines with age (Hasher and Zacks, 1988). Studies found that stressful and traumatic experiences impaired inhibitory control (Covey et al., 2013; Roos et al., 2017; Herd et al., 2018; Melara et al., 2018). Therefore, WE may have more difficulty in suppressing distractors and exhibit a lower search efficiency than NWE. For the mnemonic processing, WE showed lower accuracies and discriminability in memorizing sad and angry faces but not happy faces compared with NWE, suggesting that the deficits in working memory were restricted to negative emotions. More detailed, such deficits were evident only when the depression/state anxiety levels of individuals were high. From the simple slope analysis, we may find that the mnemonic processing increased with the level of emotional problems among NWE, which was the main reason for the moderation effects. Such a pattern of performance could be explained by the cognitive-behavioral model that the WE with high anxiety/depression have distorted or dysfunctional schemas that can distort information processing and thus were more likely to have biased working memory to sad and angry faces. A lot of studies demonstrated that depressed individuals exhibited deficits in removing/inhibiting negative stimuli (e.g., negative words) from working memory (Joormann, 2004; Joormann and Gotlib, 2008; Yoon et al., 2014) and had difficulties in disengaging sad faces from working memory (Levens and Gotlib, 2010; Foland-Ross et al., 2013), resulting in higher accuracy in working memory for negative pictures (Linden et al., 2011; Li et al., 2018). A functional MRI (fMRI) study with the *n*-back task showed greater activations elicited by negative emotional stimuli in left DLPFC among patients with depression (Kerestes et al., 2012). Therefore, the better performance of NWE on the memory for sad and angry faces may primarily result from their depression or state anxiety.

However, the working memory for negative faces among WE was less affected by their emotional problems compared with NWE, which was inconsistent with the cognitive-behavioral model. This result may stem from the impairment of working memory among WE. First, widowhood might impair working memory. Studies revealed that spousal bereavement was associated with declines in working memory among the elderly (Atalay and Staneva, 2020; Zhao et al., 2021). Second, emotional problems might also impair working memory. Previous studies revealed that individuals with a negative emotional state (e.g., depression and anxiety) performed poorly on working memory (Nebes et al., 2000; Lavric et al., 2003; Harvey et al., 2004; Christopher and MacDonald, 2005; Rose and Ebmeier, 2006; Shackman et al., 2006; Spachtholz et al., 2014; Moran, 2016; Guo et al., 2020), while positive mood could enhance working memory capacity (Storbeck and Maswood, 2016). Electrophysiological evidence further indicated that the negative emotional state led to reduced P3b amplitude at the encoding and retrieval phases of working memory (Li et al., 2010). Taken together, the working memory is dysfunctional among WE and may not be sensitive anymore to emotional problems.

### Limitations

Although the effects of age and sex were controlled statistically in this study, we still know less about the impact of age and sex on our results. First, studies with stricter control are required. Second, as there was evidence showing moderation effects of sex and age on the impact of spousal bereavement on memory (Rosnick et al., 2010), the interaction effects of age, sex, and spousal bereavement among the elderly should be further examined.

Besides, the faces adopted in this study were mainly the faces of young adults. A previous study found that the elderly showed attentional bias to own-age face cues regardless of the emotional valence, which was similar to our results (Noh and Isaacowitz, 2011). However, whether the properties of faces could affect cognitive processing remains to be investigated.

## Conclusion

Widowed elderly had a general deficit in search efficiency across emotional types, while they showed mnemonic deficits in negative faces but not positive faces. Furthermore, the level of depression or state anxiety of the elderly moderated the effects of widowhood on the deficits of mnemonic processing, i.e., the deficits were only evident among WE with a high level of depression or state anxiety. These findings reveal the attentional deficits in sad, angry, and happy faces, and the mnemonic deficits in sad and angry faces among elderly who suffer from widowhood, and point out the important role of emotional problems such as depression and state anxiety in modulating these emotional cognitive deficits.

## Data Availability Statement

The raw data supporting the conclusions of this article will be made available by the authors, without undue reservation.

## Ethics Statement

The studies involving human participants were reviewed and approved by the Ethics Committee for Human Research at Zunyi Medical University. The patients/participants provided their written informed consent to participate in this study.

## Author Contributions

TB: conception, design, acquisition of data, analysis of data, interpretation of data, funding acquisition, writing–original draft, and writing—review and editing. HK: conception, design, data acquisition, data analysis, data interpretation, funding acquisition, and writing—review and editing. YK: conception, design, acquisition of data, and writing—review and editing. BS: conception, design, and writing—review and editing. All authors contributed to the article and approved the submitted version.

## Conflict of Interest

The authors declare that the research was conducted in the absence of any commercial or financial relationships that could be construed as a potential conflict of interest.

## Publisher’s Note

All claims expressed in this article are solely those of the authors and do not necessarily represent those of their affiliated organizations, or those of the publisher, the editors and the reviewers. Any product that may be evaluated in this article, or claim that may be made by its manufacturer, is not guaranteed or endorsed by the publisher.

## Footnotes

1
https://www.pstnet.com

2
www.ibm.com/analytics/spss-statistics-software
