# Supplementary material for: Widowhood Impairs Emotional Cognition Among Elderly
Source: Front Aging Neurosci. 2022 Jan 31;13:808885. doi: 10.3389/fnagi.2021.808885 (PMC8841410; doi:10.3389/fnagi.2021.808885)
Supplement: Supplementary file 1 [file Table_1.DOCX]

Table S1 Results from visual search experiment (Mean(SD))

|  | Set size | Facial emotion | WE (N=44) | NWE (N=44) |
| --- | --- | --- | --- | --- |
| Accuracy |  |  |  |  |
|  | 2 | Sadness | 0.82(0.13) | 0.79(0.12) |
|  | 2 | Anger | 0.78(0.12) | 0.79(0.11) |
|  | 2 | Happiness | 0.98(0.05) | 0.96(0.08) |
|  | 8 | Sadness | 0.75(0.15) | 0.72(0.15) |
|  | 8 | Anger | 0.69(0.14) | 0.71(0.14) |
|  | 8 | Happiness | 0.95(0.07) | 0.91(0.13) |
| RT(ms) |  |  |  |  |
|  | 2 | Sadness | 2627.96(1101.09) | 2674.13(1273.29) |
|  | 2 | Anger | 2869.24(1543.25) | 2602.05(1320.92) |
|  | 2 | Happiness | 2162.54(909.81) | 1914.83(802.51) |
|  | 8 | Sadness | 4351.27(1988.50) | 3606.70(1283.40) |
|  | 8 | Anger | 4304.48(1627.34) | 3616.08(1387.34) |
|  | 8 | Happiness | 3357.89(1636.84) | 2689.11(1017.75) |
| Search slope |  |  |  |  |
|  | - | Sadness | 287.22(300.99) | 155.43(195.78) |
|  | - | Anger | 239.21(238.76) | 169.01(173.97) |
|  | - | Happiness | 199.22(284.69) | 129.05(139.74) |

WE: widowed elderly; NWE: non-widowed elderly.

Table S2 Results from DMTS experiment (Mean(SD))

|  | Facial emotion | WE (N=44) | NWE (N=44) |
| --- | --- | --- | --- |
| Accuracy |  |  |  |
|  | Sadness | 0.55(0.10) | 0.59(0.12) |
|  | Anger | 0.55(0.11) | 0.59(0.12) |
|  | Happiness | 0.55(0.09) | 0.54(0.10) |
| RT(ms) |  |  |  |
|  | Sadness | 2335.04(1130.04) | 2827.87(1783.57) |
|  | Anger | 3002.43(1887.42) | 2917.74(1749.86) |
|  | Happiness | 2394.99(1111.05) | 2634.63(1472.42) |
| d’ |  |  |  |
|  | Sadness | 0.35(0.59) | 0.56(0.75) |
|  | Anger | 0.31(0.63) | 0.58(0.66) |
|  | Happiness | 0.27(0.50) | 0.24(0.58) |

WE: widowed elderly; NWE: non-widowed elderly.
